# Supplementary material for: Pyruvate Dehydrogenase Kinase 1 inhibition mediated oxidative phosphorylation enhancement in cartilage promotes osteoarthritis progression
Source: BMC Musculoskelet Disord. 2023 Jul 20;24:597. doi: 10.1186/s12891-023-06585-6 (PMC10357736; doi:10.1186/s12891-023-06585-6)
Supplement: Supplementary file 1 — Additional file 1. [file 12891_2023_6585_MOESM1_ESM.zip › BP.Gsea.1653623667859/GOBP_B_CELL_MEDIATED_IMMUNITY.html]

Details for gene set GOBP\_B\_CELL\_MEDIATED\_IMMUNITY[GSEA]

|  || Dataset | 55457\_collapsed\_to\_symbols.cls.cls #OA\_versus\_normal.cls.cls #OA\_versus\_normal\_repos |
| Phenotype | cls.cls#OA\_versus\_normal\_repos |
| Upregulated in class | OA |
| GeneSet | GOBP\_B\_CELL\_MEDIATED\_IMMUNITY |
| Enrichment Score (ES) | 0.28946295 |
| Normalized Enrichment Score (NES) | 1.4397452 |
| Nominal p-value | 0.008720931 |
| FDR q-value | 0.4436058 |
| FWER p-Value | 1.0 |
Table: GSEA Results Summary

  

Fig 1: Enrichment plot: GOBP\_B\_CELL\_MEDIATED\_IMMUNITY      
 Profile of the Running ES Score & Positions of GeneSet Members on the Rank Ordered List

  

| SYMBOL | TITLE | RANK IN GENE LIST | RANK METRIC SCORE | RUNNING ES | CORE ENRICHMENT || 1 | TREM2 | triggering receptor expressed on myeloid cells 2 [Source:HGNC Symbol;Acc:HGNC:17761] | 342 | 0.726 | -0.0097 | Yes |
| 2 | TREX1 | three prime repair exonuclease 1 [Source:HGNC Symbol;Acc:HGNC:12269] | 393 | 0.709 | 0.0031 | Yes |
| 3 | CFI | complement factor I [Source:HGNC Symbol;Acc:HGNC:5394] | 455 | 0.680 | 0.0144 | Yes |
| 4 | MLH1 | mutL homolog 1 [Source:HGNC Symbol;Acc:HGNC:7127] | 497 | 0.660 | 0.0267 | Yes |
| 5 | IGHG1 | immunoglobulin heavy constant gamma 1 (G1m marker) [Source:HGNC Symbol;Acc:HGNC:5525] | 607 | 0.621 | 0.0328 | Yes |
| 6 | C4A | complement C4A (Rodgers blood group) [Source:HGNC Symbol;Acc:HGNC:1323] | 632 | 0.611 | 0.0453 | Yes |
| 7 | FCGR1A | Fc fragment of IgG receptor Ia [Source:HGNC Symbol;Acc:HGNC:3613] | 633 | 0.610 | 0.0597 | Yes |
| 8 | HLA-DMB | "major histocompatibility complex, class II, DM beta [Source:HGNC Symbol;Acc:HGNC:4935]" | 679 | 0.598 | 0.0703 | Yes |
| 9 | MSH2 | mutS homolog 2 [Source:HGNC Symbol;Acc:HGNC:7325] | 739 | 0.584 | 0.0795 | Yes |
| 10 | TNFSF13 | TNF superfamily member 13 [Source:HGNC Symbol;Acc:HGNC:11928] | 787 | 0.572 | 0.0893 | Yes |
| 11 | BTK | Bruton tyrosine kinase [Source:HGNC Symbol;Acc:HGNC:1133] | 889 | 0.545 | 0.0942 | Yes |
| 12 | C8B | complement C8 beta chain [Source:HGNC Symbol;Acc:HGNC:1353] | 1001 | 0.517 | 0.0977 | Yes |
| 13 | IGHV3-72 | immunoglobulin heavy variable 3-72 [Source:HGNC Symbol;Acc:HGNC:5622] | 1062 | 0.504 | 0.1049 | Yes |
| 14 | TRDC | T cell receptor delta constant [Source:HGNC Symbol;Acc:HGNC:12253] | 1100 | 0.496 | 0.1137 | Yes |
| 15 | NDFIP1 | Nedd4 family interacting protein 1 [Source:HGNC Symbol;Acc:HGNC:17592] | 1183 | 0.480 | 0.1186 | Yes |
| 16 | IL13RA2 | interleukin 13 receptor subunit alpha 2 [Source:HGNC Symbol;Acc:HGNC:5975] | 1202 | 0.476 | 0.1284 | Yes |
| 17 | IGLC2 | immunoglobulin lambda constant 2 [Source:HGNC Symbol;Acc:HGNC:5856] | 1210 | 0.474 | 0.1390 | Yes |
| 18 | HLA-DQA1 | "major histocompatibility complex, class II, DQ alpha 1 [Source:HGNC Symbol;Acc:HGNC:4942]" | 1334 | 0.452 | 0.1401 | Yes |
| 19 | IGHA1 | immunoglobulin heavy constant alpha 1 [Source:HGNC Symbol;Acc:HGNC:5478] | 1426 | 0.439 | 0.1433 | Yes |
| 20 | TRBC1 | T cell receptor beta constant 1 [Source:HGNC Symbol;Acc:HGNC:12156] | 1458 | 0.435 | 0.1511 | Yes |
| 21 | CD27 | CD27 molecule [Source:HGNC Symbol;Acc:HGNC:11922] | 1473 | 0.432 | 0.1602 | Yes |
| 22 | IGKC | immunoglobulin kappa constant [Source:HGNC Symbol;Acc:HGNC:5716] | 1477 | 0.432 | 0.1702 | Yes |
| 23 | IGHV3-33 | immunoglobulin heavy variable 3-33 [Source:HGNC Symbol;Acc:HGNC:5596] | 1511 | 0.427 | 0.1777 | Yes |
| 24 | HLA-DQB2 | "major histocompatibility complex, class II, DQ beta 2 [Source:HGNC Symbol;Acc:HGNC:4945]" | 1534 | 0.423 | 0.1859 | Yes |
| 25 | CD55 | CD55 molecule (Cromer blood group) [Source:HGNC Symbol;Acc:HGNC:2665] | 1619 | 0.411 | 0.1890 | Yes |
| 26 | SUSD4 | sushi domain containing 4 [Source:HGNC Symbol;Acc:HGNC:25470] | 1764 | 0.389 | 0.1869 | Yes |
| 27 | HLA-DMA | "major histocompatibility complex, class II, DM alpha [Source:HGNC Symbol;Acc:HGNC:4934]" | 1769 | 0.388 | 0.1958 | Yes |
| 28 | AICDA | activation induced cytidine deaminase [Source:HGNC Symbol;Acc:HGNC:13203] | 1825 | 0.381 | 0.2004 | Yes |
| 29 | MBL2 | mannose binding lectin 2 [Source:HGNC Symbol;Acc:HGNC:6922] | 1856 | 0.379 | 0.2070 | Yes |
| 30 | TRAF3IP2 | TRAF3 interacting protein 2 [Source:HGNC Symbol;Acc:HGNC:1343] | 1939 | 0.368 | 0.2093 | Yes |
| 31 | FCGR2B | Fc fragment of IgG receptor IIb [Source:HGNC Symbol;Acc:HGNC:3618] | 2016 | 0.360 | 0.2118 | Yes |
| 32 | CLU | clusterin [Source:HGNC Symbol;Acc:HGNC:2095] | 2083 | 0.352 | 0.2149 | Yes |
| 33 | CCR6 | C-C motif chemokine receptor 6 [Source:HGNC Symbol;Acc:HGNC:1607] | 2095 | 0.350 | 0.2223 | Yes |
| 34 | TNF | tumor necrosis factor [Source:HGNC Symbol;Acc:HGNC:11892] | 2098 | 0.350 | 0.2304 | Yes |
| 35 | HLA-DOB | "major histocompatibility complex, class II, DO beta [Source:HGNC Symbol;Acc:HGNC:4937]" | 2147 | 0.344 | 0.2348 | Yes |
| 36 | PRKCD | protein kinase C delta [Source:HGNC Symbol;Acc:HGNC:9399] | 2237 | 0.335 | 0.2357 | Yes |
| 37 | IGHM | immunoglobulin heavy constant mu [Source:HGNC Symbol;Acc:HGNC:5541] | 2241 | 0.335 | 0.2433 | Yes |
| 38 | IGHV3-21 | immunoglobulin heavy variable 3-21 [Source:HGNC Symbol;Acc:HGNC:5586] | 2404 | 0.314 | 0.2380 | Yes |
| 39 | IGHV1-69 | immunoglobulin heavy variable 1-69 [Source:HGNC Symbol;Acc:HGNC:5558] | 2461 | 0.307 | 0.2409 | Yes |
| 40 | TGFB1 | transforming growth factor beta 1 [Source:HGNC Symbol;Acc:HGNC:11766] | 2490 | 0.303 | 0.2459 | Yes |
| 41 | CR2 | complement C3d receptor 2 [Source:HGNC Symbol;Acc:HGNC:2336] | 2584 | 0.292 | 0.2455 | Yes |
| 42 | LIG4 | DNA ligase 4 [Source:HGNC Symbol;Acc:HGNC:6601] | 2668 | 0.283 | 0.2456 | Yes |
| 43 | IGHV4-61 | immunoglobulin heavy variable 4-61 [Source:HGNC Symbol;Acc:HGNC:5655] | 2722 | 0.278 | 0.2480 | Yes |
| 44 | FCER1G | Fc fragment of IgE receptor Ig [Source:HGNC Symbol;Acc:HGNC:3611] | 2752 | 0.275 | 0.2522 | Yes |
| 45 | CD19 | CD19 molecule [Source:HGNC Symbol;Acc:HGNC:1633] | 2757 | 0.274 | 0.2584 | Yes |
| 46 | C3 | complement C3 [Source:HGNC Symbol;Acc:HGNC:1318] | 2764 | 0.273 | 0.2644 | Yes |
| 47 | IGHV3-7 | immunoglobulin heavy variable 3-7 [Source:HGNC Symbol;Acc:HGNC:5620] | 2798 | 0.270 | 0.2682 | Yes |
| 48 | HLA-DQB1 | "major histocompatibility complex, class II, DQ beta 1 [Source:HGNC Symbol;Acc:HGNC:4944]" | 2840 | 0.266 | 0.2712 | Yes |
| 49 | C1QA | complement C1q A chain [Source:HGNC Symbol;Acc:HGNC:1241] | 2902 | 0.260 | 0.2726 | Yes |
| 50 | ERCC1 | "ERCC excision repair 1, endonuclease non-catalytic subunit [Source:HGNC Symbol;Acc:HGNC:3433]" | 2948 | 0.255 | 0.2751 | Yes |
| 51 | IGHV3-73 | immunoglobulin heavy variable 3-73 [Source:HGNC Symbol;Acc:HGNC:5623] | 3082 | 0.241 | 0.2704 | Yes |
| 52 | C1QBP | complement C1q binding protein [Source:HGNC Symbol;Acc:HGNC:1243] | 3083 | 0.241 | 0.2760 | Yes |
| 53 | INPP5D | inositol polyphosphate-5-phosphatase D [Source:HGNC Symbol;Acc:HGNC:6079] | 3085 | 0.241 | 0.2817 | Yes |
| 54 | NOD2 | nucleotide binding oligomerization domain containing 2 [Source:HGNC Symbol;Acc:HGNC:5331] | 3154 | 0.235 | 0.2819 | Yes |
| 55 | IL27RA | interleukin 27 receptor subunit alpha [Source:HGNC Symbol;Acc:HGNC:17290] | 3298 | 0.221 | 0.2759 | Yes |
| 56 | SHLD2 | shieldin complex subunit 2 [Source:HGNC Symbol;Acc:HGNC:28773] | 3300 | 0.221 | 0.2810 | Yes |
| 57 | C1QB | complement C1q B chain [Source:HGNC Symbol;Acc:HGNC:1242] | 3365 | 0.215 | 0.2811 | Yes |
| 58 | TRBC2 | T cell receptor beta constant 2 [Source:HGNC Symbol;Acc:HGNC:12157] | 3401 | 0.211 | 0.2833 | Yes |
| 59 | PTPN6 | protein tyrosine phosphatase non-receptor type 6 [Source:HGNC Symbol;Acc:HGNC:9658] | 3434 | 0.208 | 0.2857 | Yes |
| 60 | IGHV3-23 | immunoglobulin heavy variable 3-23 [Source:HGNC Symbol;Acc:HGNC:5588] | 3449 | 0.207 | 0.2895 | Yes |
| 61 | TLR8 | toll like receptor 8 [Source:HGNC Symbol;Acc:HGNC:15632] | 3579 | 0.196 | 0.2840 | No |
| 62 | FCER2 | Fc fragment of IgE receptor II [Source:HGNC Symbol;Acc:HGNC:3612] | 3785 | 0.178 | 0.2721 | No |
| 63 | XCL1 | X-C motif chemokine ligand 1 [Source:HGNC Symbol;Acc:HGNC:10645] | 3789 | 0.178 | 0.2761 | No |
| 64 | C8A | complement C8 alpha chain [Source:HGNC Symbol;Acc:HGNC:1352] | 3918 | 0.163 | 0.2699 | No |
| 65 | C7 | complement C7 [Source:HGNC Symbol;Acc:HGNC:1346] | 4078 | 0.148 | 0.2609 | No |
| 66 | IL9R | interleukin 9 receptor [Source:HGNC Symbol;Acc:HGNC:6030] | 4118 | 0.144 | 0.2612 | No |
| 67 | NBN | nibrin [Source:HGNC Symbol;Acc:HGNC:7652] | 4136 | 0.143 | 0.2633 | No |
| 68 | HLA-DOA | "major histocompatibility complex, class II, DO alpha [Source:HGNC Symbol;Acc:HGNC:4936]" | 4344 | 0.125 | 0.2500 | No |
| 69 | ZP3 | zona pellucida glycoprotein 3 [Source:HGNC Symbol;Acc:HGNC:13189] | 4367 | 0.123 | 0.2512 | No |
| 70 | C5 | complement C5 [Source:HGNC Symbol;Acc:HGNC:1331] | 4387 | 0.122 | 0.2526 | No |
| 71 | IRF7 | interferon regulatory factor 7 [Source:HGNC Symbol;Acc:HGNC:6122] | 4411 | 0.120 | 0.2536 | No |
| 72 | CD28 | CD28 molecule [Source:HGNC Symbol;Acc:HGNC:1653] | 4543 | 0.110 | 0.2459 | No |
| 73 | CD46 | CD46 molecule [Source:HGNC Symbol;Acc:HGNC:6953] | 4563 | 0.108 | 0.2470 | No |
| 74 | FCGR3A | Fc fragment of IgG receptor IIIa [Source:HGNC Symbol;Acc:HGNC:3619] | 4594 | 0.105 | 0.2471 | No |
| 75 | IGHV4-34 | immunoglobulin heavy variable 4-34 [Source:HGNC Symbol;Acc:HGNC:5650] | 4746 | 0.091 | 0.2374 | No |
| 76 | IL21R | interleukin 21 receptor [Source:HGNC Symbol;Acc:HGNC:6006] | 4810 | 0.087 | 0.2345 | No |
| 77 | RNF8 | ring finger protein 8 [Source:HGNC Symbol;Acc:HGNC:10071] | 4938 | 0.077 | 0.2264 | No |
| 78 | TP53BP1 | tumor protein p53 binding protein 1 [Source:HGNC Symbol;Acc:HGNC:11999] | 4946 | 0.076 | 0.2276 | No |
| 79 | ATAD5 | ATPase family AAA domain containing 5 [Source:HGNC Symbol;Acc:HGNC:25752] | 5020 | 0.071 | 0.2236 | No |
| 80 | CRP | C-reactive protein [Source:HGNC Symbol;Acc:HGNC:2367] | 5154 | 0.059 | 0.2145 | No |
| 81 | HMCES | "5-hydroxymethylcytosine binding, ES cell specific [Source:HGNC Symbol;Acc:HGNC:24446]" | 5402 | 0.040 | 0.1961 | No |
| 82 | C9 | complement C9 [Source:HGNC Symbol;Acc:HGNC:1358] | 5415 | 0.039 | 0.1961 | No |
| 83 | HLA-DPB1 | "major histocompatibility complex, class II, DP beta 1 [Source:HGNC Symbol;Acc:HGNC:4940]" | 5486 | 0.033 | 0.1914 | No |
| 84 | CD40LG | CD40 ligand [Source:HGNC Symbol;Acc:HGNC:11935] | 5529 | 0.030 | 0.1888 | No |
| 85 | IL2 | interleukin 2 [Source:HGNC Symbol;Acc:HGNC:6001] | 5819 | 0.007 | 0.1663 | No |
| 86 | TNFSF4 | TNF superfamily member 4 [Source:HGNC Symbol;Acc:HGNC:11934] | 5930 | 0.000 | 0.1577 | No |
| 87 | UNG | uracil DNA glycosylase [Source:HGNC Symbol;Acc:HGNC:12572] | 6078 | -0.011 | 0.1464 | No |
| 88 | KMT5B | lysine methyltransferase 5B [Source:HGNC Symbol;Acc:HGNC:24283] | 6198 | -0.020 | 0.1376 | No |
| 89 | CD74 | CD74 molecule [Source:HGNC Symbol;Acc:HGNC:1697] | 6374 | -0.033 | 0.1246 | No |
| 90 | IGHV5-51 | immunoglobulin heavy variable 5-51 [Source:HGNC Symbol;Acc:HGNC:5659] | 6399 | -0.035 | 0.1236 | No |
| 91 | HLA-DRA | "major histocompatibility complex, class II, DR alpha [Source:HGNC Symbol;Acc:HGNC:4947]" | 6550 | -0.046 | 0.1129 | No |
| 92 | MYD88 | MYD88 innate immune signal transduction adaptor [Source:HGNC Symbol;Acc:HGNC:7562] | 6638 | -0.052 | 0.1073 | No |
| 93 | CARD9 | caspase recruitment domain family member 9 [Source:HGNC Symbol;Acc:HGNC:16391] | 6645 | -0.053 | 0.1081 | No |
| 94 | IL4 | interleukin 4 [Source:HGNC Symbol;Acc:HGNC:6014] | 6673 | -0.054 | 0.1072 | No |
| 95 | CD40 | CD40 molecule [Source:HGNC Symbol;Acc:HGNC:11919] | 6825 | -0.065 | 0.0969 | No |
| 96 | IGHV3-20 | immunoglobulin heavy variable 3-20 [Source:HGNC Symbol;Acc:HGNC:5585] | 6859 | -0.068 | 0.0959 | No |
| 97 | FOXP3 | forkhead box P3 [Source:HGNC Symbol;Acc:HGNC:6106] | 7035 | -0.082 | 0.0842 | No |
| 98 | HLA-DRB1 | "major histocompatibility complex, class II, DR beta 1 [Source:HGNC Symbol;Acc:HGNC:4948]" | 7087 | -0.086 | 0.0822 | No |
| 99 | TBX21 | T-box transcription factor 21 [Source:HGNC Symbol;Acc:HGNC:11599] | 7207 | -0.097 | 0.0752 | No |
| 100 | SERPING1 | serpin family G member 1 [Source:HGNC Symbol;Acc:HGNC:1228] | 7286 | -0.101 | 0.0714 | No |
| 101 | C6 | complement C6 [Source:HGNC Symbol;Acc:HGNC:1339] | 7316 | -0.103 | 0.0716 | No |
| 102 | RIF1 | replication timing regulatory factor 1 [Source:HGNC Symbol;Acc:HGNC:23207] | 7448 | -0.114 | 0.0640 | No |
| 103 | CD70 | CD70 molecule [Source:HGNC Symbol;Acc:HGNC:11937] | 7491 | -0.118 | 0.0635 | No |
| 104 | IGLL1 | immunoglobulin lambda like polypeptide 1 [Source:HGNC Symbol;Acc:HGNC:5870] | 7544 | -0.122 | 0.0623 | No |
| 105 | TCIRG1 | "T cell immune regulator 1, ATPase H+ transporting V0 subunit a3 [Source:HGNC Symbol;Acc:HGNC:11647]" | 7600 | -0.127 | 0.0610 | No |
| 106 | NSD2 | nuclear receptor binding SET domain protein 2 [Source:HGNC Symbol;Acc:HGNC:12766] | 7622 | -0.129 | 0.0624 | No |
| 107 | C1RL | complement C1r subcomponent like [Source:HGNC Symbol;Acc:HGNC:21265] | 7656 | -0.131 | 0.0629 | No |
| 108 | C1R | complement C1r [Source:HGNC Symbol;Acc:HGNC:1246] | 7716 | -0.135 | 0.0614 | No |
| 109 | PTPRC | protein tyrosine phosphatase receptor type C [Source:HGNC Symbol;Acc:HGNC:9666] | 7744 | -0.137 | 0.0626 | No |
| 110 | C4BPB | complement component 4 binding protein beta [Source:HGNC Symbol;Acc:HGNC:1328] | 7802 | -0.141 | 0.0614 | No |
| 111 | CSF2RB | colony stimulating factor 2 receptor subunit beta [Source:HGNC Symbol;Acc:HGNC:2436] | 7826 | -0.143 | 0.0630 | No |
| 112 | CD226 | CD226 molecule [Source:HGNC Symbol;Acc:HGNC:16961] | 7930 | -0.149 | 0.0584 | No |
| 113 | EXO1 | exonuclease 1 [Source:HGNC Symbol;Acc:HGNC:3511] | 8088 | -0.162 | 0.0500 | No |
| 114 | PAXIP1 | PAX interacting protein 1 [Source:HGNC Symbol;Acc:HGNC:8624] | 8148 | -0.167 | 0.0493 | No |
| 115 | STAT6 | signal transducer and activator of transcription 6 [Source:HGNC Symbol;Acc:HGNC:11368] | 8191 | -0.170 | 0.0500 | No |
| 116 | SUPT6H | "SPT6 homolog, histone chaperone and transcription elongation factor [Source:HGNC Symbol;Acc:HGNC:11470]" | 8752 | -0.213 | 0.0111 | No |
| 117 | CD81 | CD81 molecule [Source:HGNC Symbol;Acc:HGNC:1701] | 8827 | -0.220 | 0.0105 | No |
| 118 | MSH6 | mutS homolog 6 [Source:HGNC Symbol;Acc:HGNC:7329] | 8846 | -0.221 | 0.0143 | No |
| 119 | IL10 | interleukin 10 [Source:HGNC Symbol;Acc:HGNC:5962] | 8855 | -0.221 | 0.0189 | No |
| 120 | C8G | complement C8 gamma chain [Source:HGNC Symbol;Acc:HGNC:1354] | 8871 | -0.223 | 0.0229 | No |
| 121 | PARP3 | poly(ADP-ribose) polymerase family member 3 [Source:HGNC Symbol;Acc:HGNC:273] | 9186 | -0.246 | 0.0041 | No |
| 122 | BCL10 | BCL10 immune signaling adaptor [Source:HGNC Symbol;Acc:HGNC:989] | 9248 | -0.252 | 0.0053 | No |
| 123 | FOXJ1 | forkhead box J1 [Source:HGNC Symbol;Acc:HGNC:3816] | 9364 | -0.261 | 0.0024 | No |
| 124 | IGHD | immunoglobulin heavy constant delta [Source:HGNC Symbol;Acc:HGNC:5480] | 9396 | -0.263 | 0.0062 | No |
| 125 | HLA-DPA1 | "major histocompatibility complex, class II, DP alpha 1 [Source:HGNC Symbol;Acc:HGNC:4938]" | 9481 | -0.273 | 0.0060 | No |
| 126 | C4BPA | complement component 4 binding protein alpha [Source:HGNC Symbol;Acc:HGNC:1325] | 9505 | -0.274 | 0.0107 | No |
| 127 | LTA | lymphotoxin alpha [Source:HGNC Symbol;Acc:HGNC:6709] | 9921 | -0.313 | -0.0144 | No |
| 128 | C1S | complement C1s [Source:HGNC Symbol;Acc:HGNC:1247] | 10012 | -0.323 | -0.0139 | No |
| 129 | SWAP70 | switching B cell complex subunit SWAP70 [Source:HGNC Symbol;Acc:HGNC:17070] | 10019 | -0.323 | -0.0067 | No |
| 130 | TFRC | transferrin receptor [Source:HGNC Symbol;Acc:HGNC:11763] | 10184 | -0.339 | -0.0116 | No |
| 131 | NECTIN2 | nectin cell adhesion molecule 2 [Source:HGNC Symbol;Acc:HGNC:9707] | 10204 | -0.341 | -0.0050 | No |
| 132 | BATF | basic leucine zipper ATF-like transcription factor [Source:HGNC Symbol;Acc:HGNC:958] | 10312 | -0.352 | -0.0051 | No |
| 133 | APCS | "amyloid P component, serum [Source:HGNC Symbol;Acc:HGNC:584]" | 10326 | -0.353 | 0.0022 | No |
| 134 | IGHE | immunoglobulin heavy constant epsilon [Source:HGNC Symbol;Acc:HGNC:5522] | 10676 | -0.390 | -0.0160 | No |
| 135 | HLA-G | "major histocompatibility complex, class I, G [Source:HGNC Symbol;Acc:HGNC:4964]" | 10869 | -0.413 | -0.0213 | No |
| 136 | IL4R | interleukin 4 receptor [Source:HGNC Symbol;Acc:HGNC:6015] | 10902 | -0.417 | -0.0139 | No |
| 137 | MASP2 | MBL associated serine protease 2 [Source:HGNC Symbol;Acc:HGNC:6902] | 11001 | -0.430 | -0.0115 | No |
| 138 | HPX | hemopexin [Source:HGNC Symbol;Acc:HGNC:5171] | 11156 | -0.451 | -0.0129 | No |
| 139 | IL9 | interleukin 9 [Source:HGNC Symbol;Acc:HGNC:6029] | 11309 | -0.472 | -0.0137 | No |
| 140 | CR1 | complement C3b/C4b receptor 1 (Knops blood group) [Source:HGNC Symbol;Acc:HGNC:2334] | 11376 | -0.483 | -0.0075 | No |
| 141 | HSPD1 | heat shock protein family D (Hsp60) member 1 [Source:HGNC Symbol;Acc:HGNC:5261] | 11427 | -0.492 | 0.0002 | No |
| 142 | BCL3 | BCL3 transcription coactivator [Source:HGNC Symbol;Acc:HGNC:998] | 11431 | -0.493 | 0.0116 | No |
| 143 | HLA-E | "major histocompatibility complex, class I, E [Source:HGNC Symbol;Acc:HGNC:4962]" | 11659 | -0.526 | 0.0062 | No |
| 144 | CLCF1 | cardiotrophin like cytokine factor 1 [Source:HGNC Symbol;Acc:HGNC:17412] | 12360 | -0.695 | -0.0323 | No |
| 145 | THOC1 | THO complex 1 [Source:HGNC Symbol;Acc:HGNC:19070] | 12503 | -0.749 | -0.0258 | No |
| 146 | B2M | beta-2-microglobulin [Source:HGNC Symbol;Acc:HGNC:914] | 12507 | -0.750 | -0.0083 | No |
| 147 | HLA-DRB4 | "major histocompatibility complex, class II, DR beta 4 [Source:HGNC Symbol;Acc:HGNC:4952]" | 12636 | -0.815 | 0.0009 | No |
| 148 | BCL6 | BCL6 transcription repressor [Source:HGNC Symbol;Acc:HGNC:1001] | 12667 | -0.838 | 0.0183 | No |
Table: GSEA details [plain text format]

  

Fig 2: GOBP\_B\_CELL\_MEDIATED\_IMMUNITY      
 Blue-Pink O' Gram in the Space of the Analyzed GeneSet

  

Fig 3: GOBP\_B\_CELL\_MEDIATED\_IMMUNITY: Random ES distribution      
 Gene set null distribution of ES for **GOBP\_B\_CELL\_MEDIATED\_IMMUNITY**
